# Evidence of Small Fungal Cysteine-Rich Proteins Acting as Biosurfactants and Self-Assembling into Large Fibers

**DOI:** 10.3390/ijms241813843

**Published:** 2023-09-08

**Authors:** Rossana Pitocchi, Ilaria Stanzione, Anna Illiano, Angela Amoresano, Oreste Tarallo, Paola Cicatiello, Alessandra Piscitelli, Paola Giardina

**Affiliations:** Department of Chemical Sciences, University of Naples Federico II, Via Cintia, 80126 Naples, Italy; rossana.pitocchi@unina.it (R.P.); ilaria.stanzione@unina.it (I.S.); anna.illiano@unina.it (A.I.); angela.amoresano@unina.it (A.A.); oreste.tarallo@unina.it (O.T.); alessandra.piscitelli@unina.it (A.P.); paola.giardina@unina.it (P.G.)

**Keywords:** bioemulsifiers, functional amyloids, surface tension, marine fungi

## Abstract

Fungi produce surface-active proteins, among which hydrophobins are the most characterized and attractive also for their ability to form functional amyloids. Our most recent findings show that these abilities are shared with other classes of fungal proteins. Indeed, in this paper, we compared the characteristics of a class I hydrophobin (Vmh2 from *Pleurotus ostreatus*) and an unknown protein (named PAC3), extracted from the marine fungal strain *Acremonium sclerotigenum,* which does not belong to the same protein family based on its sequence features. They both proved to be good biosurfactants, stabilizing emulsions in several conditions (concentration, pH, and salinity) and decreasing surface tension to a comparable value to that of some synthetic surfactants. After that, we observed for both Vmh2 and PAC3 the formation of giant fibers without the need for harsh conditions or long incubation time, a remarkable ability herein reported for the first time.

## 1. Introduction

Biosurfactants (BSs) are water-soluble surface-active species, naturally occurring or synthesized from natural components. Their main properties are strong surface activity, adsorption to different interfaces and surfaces, and their ability to self-assemble into aggregates or micelles. In synthetic surfactants, the main structural characteristics are a hydrophilic group, which may be charged, and a hydrophobic group, which is usually an alkyl chain. In bioemulsifiers (BEs), the headgroup can consist of amino acids or saccharide groups, and the hydrophobic portion could be a group of hydrophobic amino acids, an alkyl chain, or other classes of compounds, i.e., the steroid-alkaloid group. The distinct separation between the hydrophilic and hydrophobic regions is often less definite in BSs as compared with synthetic surfactants. Low molecular weight BSs are typically glycolipids or lipopetides and are usually produced by bacteria [1]. The higher molecular weight BSs are often biopolymers, such as polysaccharides, proteins, liposaccharides, or lipoproteins, and are mainly extracellular. They should be more appropriately defined as BEs because they can emulsify two immiscible liquids, even at low concentrations, while not significantly reducing surface or interfacial tension [2].

The wide applications of BEs and BSs in several areas, such as food, agriculture, chemicals, medicine, and energy, have led scientific studies to disclose novel routes to optimize production parameters, increase yields, and cut costs in order to find applications of these compounds at the industrial level. Among other microorganisms, fungi are gaining global interest for their ability to produce these biopolymers at high concentrations under relatively simple production conditions [3].

In particular, filamentous fungi produce and secrete small, amphipathic proteins called hydrophobins (HPBs) (~70–150 residues). HPBs are highly surface-active proteins due to their asymmetrical distribution of hydrophilic and hydrophobic amino acids on a compact globular protein surface [4]. They spontaneously self-assemble at hydrophobic: hydrophilic interfaces into amphipathic layers, which are functional for fungi because they allow them to breach air and water boundaries; otherwise, surface tension could impede their growth. The structural feature common to all HPBs is the presence of eight conserved cysteine residues that form four disulfide bridges, which connect C1–C6, C2–C5, C3–C4, and C7–C8. The HPB family has been divided into two classes based on the stability of the layer they form and the length of the inter-cysteine spaces [5,6]. Their amino acid sequences show low similarity even when they belong to the same class. For instance, class I hydrophobins share as little as 10% identity in their alignments despite belonging to the same protein family. On the other hand, class II hydrophobins share more sequence identity (48%) and identical cysteine residue spacing [7].

Class I HPBs, produced by ascomycetes and basidiomycetes species, assemble into insoluble layers, which share the cross-β structure and morphology with amyloid fibrils. These extremely stable layers can only be solubilized with harsh acid treatments (formic acid or trifluoroacetic acid) [6]. On the other hand, class II HPBs, produced only by ascomycetes, form aggregates soluble in detergents or alcohols [8]. More recently, this classification has been questioned. Indeed, an intermediate third class has been proposed based on the analysis of hydrophobicity profiles and protein properties [9]. Moreover, a further subdivision of class I HPBs between ascomycete and basidiomycete (class IA and IB, respectively) has been introduced [10].

Class I HPBs are among the first proteins recognized as functional amyloids [11]. These ‘functional amyloids’ can play physiological roles, being involved in a significant variety of processes, including storage, structural scaffolding, cell signaling, biofilm formation, stabilization, and more.

We studied the self-assembly of the class I HPB Vmh2 from the basidiomycete *Pleurotus ostreatus* in aqueous solutions by using different spectroscopic and microscopic techniques. Vmh2 spontaneously self-assembles into isolated, long, and twisted amyloid fibrils. This process is promoted by acidic pH, temperature, and Ca^2+^ ions [12]. Several applications of this protein, alone or fused with other biotechnologically interesting proteins, have been developed [6]. Furthermore, our research group extracted new putative HFBs from selected marine fungi, since the unique environmental conditions in which they grow lead to the development of proteomes different from those of their terrestrial counterparts, providing new and intriguing biological products [13]. Six HPB-like proteins have been partially isolated and characterized, and their ability to change the wettability of a hydrophobic surface and to stabilize water/oil emulsions have been assessed, suggesting their potential as BS or BE [14]. In particular, a protein isolated from *Acremonium sclerotigenum*, named PAC3, showed a high propensity to form an emulsion with olive oil, form amyloid fibrils, and interact with hydrophobic and hydrophilic surfaces. For these characteristics, PAC3 has been previously classified as class I HPB [15]. 

In this paper, we analyze the amino acid sequence of PAC3 and observe that it does not belong to the class I HPB, as Vmh2. Nevertheless, we demonstrate that these two proteins can form giant fibers without needing an acidic pH, high temperature, and long incubation time. Moreover, the BS and BE properties of Vmh2 were herein studied for the first time and compared with those of PAC3. 

## 2. Results

Two filamentous fungi, *P. ostreatus* and *A. sclerotigenum*, a terrestrial and a marine strain, are already known to produce amphiphilic self-assembling proteins, Vmh2 and PAC3, respectively. The former is a class I HPB, and the latter, PAC3, shares many characteristics with the HPB proteins family; nonetheless, its sequence is unknown. 

### 2.1. PAC3 Sequence Analysis 

**Figure 1:**
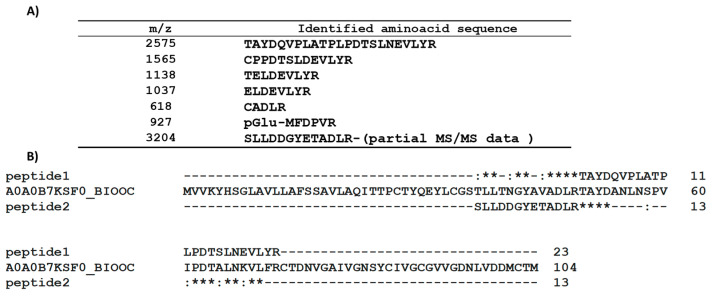
(**A**) Table of the identified amino acid sequences from MALDI-TOF analysis; (**B**) alignment of peptide 1 (2575 *m*/*z*) and peptide 2 (3204 *m*/*z*) with the unknown protein from *B. ochroleuca* resulting from the BLAST research in the Uniprot database. (*) conserved sequence (identical); (:) conservative mutation; (-) Gap.

The *A. sclerotigenum* genome sequence is unknown; thus, several experiments were performed to determine the de novo amino acid sequencing of PAC3. No results about the N-terminal sequence were found using the Edman approach, indicating a blocked N-terminus. Classical trypsin hydrolysis protocols, involving denaturation, reduction, and carboxyamidomethylation, did not produce any detectable peptides, neither in solution nor in situ (SDS-PAGE band extraction), probably due to rigid protein conformation that limits the proteolytic action of the digestive enzyme, despite the denaturing environment. Only when the protein was pretreated with 5 mM of dithiothreitol (DTT) at 60 °C for 10 min, it was efficiently denatured and hydrolyzed. The peptide mixture was analyzed by using MALDI-TOF, and the spectrum was recorded in the 400–3500 *m*/*z* mass range. It was characterized by the presence of a single intense base peak at 2575 *m*/*z* and a limited number of other low signals reported in Figure 1. The MALDI-MS/MS analysis of the fragmentation spectra led to the sequences summarized in Table S1. The fragmentation spectrum of the peptide at 2575 *m*/*z* is reported as an example in Figure S2. It can be observed that the 927 *m*/*z* signal could be associated with the N-terminus of the protein (Q as the first amino acid) and that only a small portion of the sequence corresponding to the 3204 *m*/*z* signal was reconstructed. 

Homology research regarding the sequence of the peptide at 2575 *m*/*z* using the BLAST tool from Uniprot revealed a partial identity with an unknown protein from the fungus *Bionectria ochroleuca* (A0A0B7K0S3_BIOOC), which belongs to the same order (Hypocreales) of *A. sclerotigeum*. Moreover, the partial sequence determined for the 3204 *m*/*z* peptide can be effectively aligned to the same protein, using the ClustalW tool, resulting in placement just before the sequence of the peptide at 2575 *m*/*z* (Figure 1). The obtained result showed a 44% identity percentage with the A0A0B7K0S3_BIOOC protein in the aligned region.

MALDI-TOF analysis of the reduced and carboxyamidomethylated protein compared to the non-treated one showed an increase in the molecular weight corresponding to the presence of six cysteines (Figure S3), analogously to the protein A0A0B7K0S3_BIOOC. However, it differs significantly from the HPBs family, which exhibits a conserved pattern of eight cysteines. 

### 2.2. Self-Assembling of Protein into Fibers

Vmh2, being a class I HPB, forms amyloid fibrils spontaneously. Surprisingly, the small Cys-rich protein PAC3, herein found to not belong to this class, shows the same ability to self-assemble into fibrils [15].

**Figure 2:**
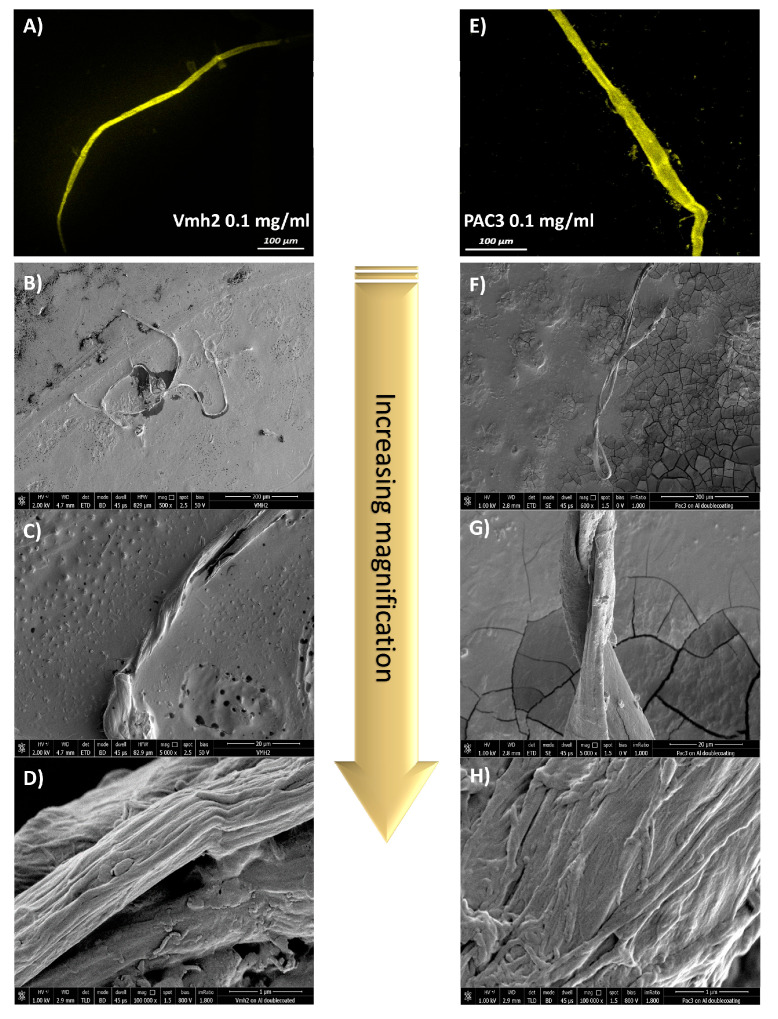
Confocal laser microscope images of Vmh2 (**A**) and PAC3 (**E**) amyloid fibers stained by ThT. A total of 20 µL of protein solutions containing 3 µM ThT were deposited on a polystyrene support and air-dried prior to visualization. SEM images of Vmh2 (**B**–**D**) and PAC3 (**F**–**H**) fibers at different magnifications of 600×, 5000×, and 100,000×, respectively. A total of 20 µL of protein solutions were deposited on the surfaces, air-dried, and Au-Pd-coated prior to visualization.

Moreover, the recombinant Vmh2 fused to GFP (green fluorescent protein) was recently drop-cast on a polystyrene surface and analyzed by using laser confocal microscopy, observing fluorescent giant fibers (hundreds of microns long and tens of microns wide) [16]. To understand if similar structures were also formed by the native Vmh2 and by PAC3, the Thioflavine T (ThT) fluorescent probe (commonly used to detect amyloid fibrils) was added to the protein solutions and analyzed by using a confocal microscope after drop-casting on a polystyrene surface. Again, the presence of large amyloid fibers formed by both proteins, whose width ranged from 20 to 30 µm, with a length in the order of 0.5 mm, was observed. Therefore, to gain deeper insights into the size/morphology of the fibers formed by Vmh2 and PAC3, scanning electron microscopy (SEM) experiments were carried out. SEM analysis of the structures formed by Vmh2 and PAC3 deposited either on polystyrene or aluminum revealed the presence of long, unbranched, twisted ribbon-like filaments (Figure 2, Figures S4 and S5). These filaments are often bent, twisted, or helical in shape and can vary in length and width. They are typically several hundred micrometers to millimeters in length, thus being significantly longer compared to typical fibrils [17]. Moreover, the ribbons are characterized by an average thickness of 5 ± 1 μm and an average width of 25 ± 2 μm. These ribbons seem to exhibit a hierarchical organization in which individual fibrils of nanometric dimension (average diameter around 30 nm) intertwine, align, or bundle together to form larger structures as revealed by examining high-magnification images (Figure S4). The aggregation and bundling of multiple individual fibrils can be considered by the observed rough and irregular surface texture of the ribbons.

### 2.3. Emulsification Ability

**Figure 3:**
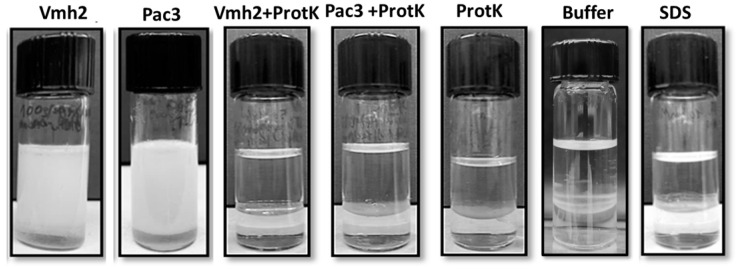
Emulsions between Dectol and protein solutions (0.1 mg/mL, 50 mM TrisHCl pH 8) 2:1 *v*/*v*. Starting from the left: emulsions formed by Vmh2, PAC3, and Vmh2 after hydrolysis; PAC3 after hydrolysis; and Proteinase K, buffer, and SDS as controls. Images were acquired after 24 h.

To investigate the performances of these proteins as emulsifying agents, hence their ability to disperse oil in water, emulsification tests were performed according to the protocol of Blesic et al. [18]. Firstly, to assess the protein’s ability to form stable emulsions, their solutions (0.1 mg/mL) were used, and their good performances as BE were verified, achieving an E_24_ of 78% and 85% for Vmh2 and PAC3, respectively. A buffer (50 mM Tris-HCl, pH 8) and 0.02 mM SDS (equivalent molar concentration to that of the proteins) were used as a negative and positive control, respectively (Figure 3). Protein emulsions were also evaluated in the long term, and they were stable for at least one month. 

A hydrolytic treatment with Proteinase K was performed to ensure that the observed emulsifying capability was due to the presence of proteins. PAC3 hydrolysis was obtained only when the protein was previously treated as described above, as confirmed by SDS-PAGE (Figure S6). The hydrolyzed proteins did not form emulsions, proving their crucial role in emulsion formation. 

Emulsions were then made in the presence of the ThT for the confocal microscopy analysis (Figure S7). Images acquired in dried conditions indicate the presence of amyloid aggregates. Moreover, their distribution lets us hypothesize the oil-in water nature of the emulsions, as reported elsewhere [18,19].

#### 2.3.1. Effect of Protein Concentrations

**Figure 4:**
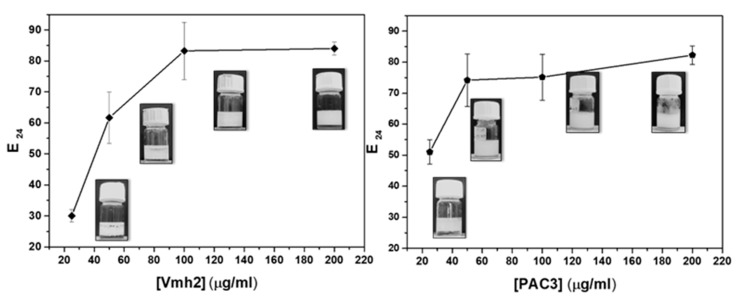
Emulsification index (E_24_) of Vmh2 and PAC3 versus protein concentrations. At each concentration, an example of the emulsion visible after 24 h is shown. Error bars in the graph represent the standard deviation calculated based on at least two biological and three technical replicates.

Tests were then conducted at different protein concentrations (0.025, 0.05, 0.1, and 0.2 mg/mL). Both proteins showed a remarkable ability to stabilize emulsions between immiscible phases. As expected, the emulsion indexes increased as the protein concentrations increased, reaching a comparable E_24_ maximum value (Figure 4). 

#### 2.3.2. Effect of pH and Salt Concentration 

**Figure 5:**
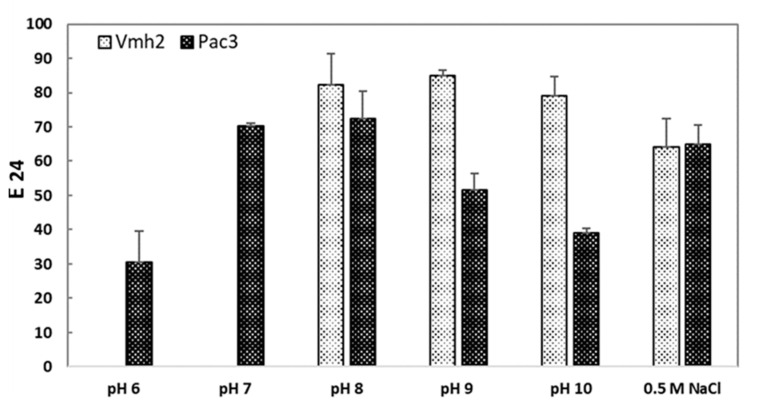
Emulsification index (E_24_) of Vmh2 and PAC3 solutions at fixed concentration (0.1 mg/mL) versus pHs and in presence of salt. Error bars in the graph represent the standard deviation calculated based on at least two biological and three technical replicates.

As previously reported, both proteins have poor solubility at an acidic pH [12,15]. More specifically, pH ≥ 7.5 is needed to completely solubilize Vmh2, while PAC3 is soluble since pH 6; thus, different ranges of pHs were tested for the two proteins. As reported in Figure 5, the emulsion efficiency of Vmh2 does not vary within the tested pH range; in contrast, PAC3 showed a maximum efficiency between pH 7 and 8. The presence of salt affected the E_24_ values of both proteins, causing their slight reduction at 0.5 M NaCl, while no emulsion at all was formed by doubling the salt concentration (Figure 5).

#### 2.3.3. Effect of Temperature

**Figure 6:**
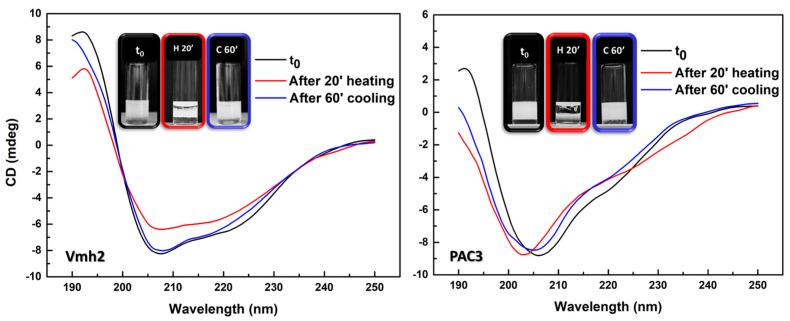
CD spectra of Vmh2 (on the left) and PAC3 (on the right) after heating at 95 °C for 20′ and after cooling at RT for 60′. Emulsions after each thermal treatment were reported as an insert for both proteins.

The effect of temperature was evaluated by exposing proteins to high temperature (95°) for 20′. Afterward, a substantial decrease in their emulsification ability was observed. However, this ability was restored after prolonged cooling at room temperature. This interesting reversible phenomenon was deepened considering possible changes in the structure of Vmh2 and PAC3, analyzed by using circular dichroism spectroscopy (Figure 6). Results showed changes in the secondary structures right after heating but their restoration (only partially in the case of PAC3) after 60′ at room temperature, highlighting the correlation between the structure and functionality of these proteins.

### 2.4. Surface Activity

**Figure 7:**
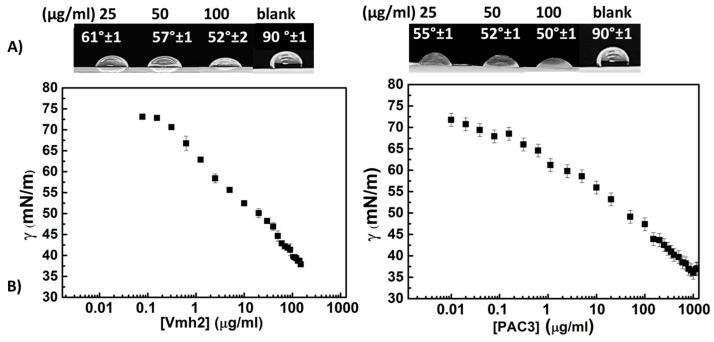
Surface activity. (**A**) Drop collapse tests of Vmh2 solution on the left, PAC3 on the right. A total of 10 µL of protein solution at increasing concentrations was deposited on Parafilm, and pictures were acquired after 10′. Contact angles were estimated using Image J version: 1.8.0. (**B**) Surface tension curves of Vmh2 and PAC3. Each protein was suspended in 50 mM Tris HCl pH 8, and curves were obtained using the Du Noüy ring method. Error bars in the graph represent the standard deviation calculated based on at least two biological and three technical replicates.

A reduction in surface tension is a fundamental criterion for surfactant characterization and was herein qualitatively measured by using the drop collapse test (Figure 7A) and more accurately using the platinum-iridium ring method (Figure 7B). Protein solutions at their maximal concentration were prepared, reaching 0.14 mg/mL for Vmh2 and 1 mg/mL for PAC3. Then, serial dilutions were carried out starting from these concentrations until the surface tension reached the γ value of the buffer solution.

The tension curves of Vmh2 and PAC3 showed a constant decrease in the surface tension values as a function of concentration without reaching a critical micellar concentration (CMC). The minimum surface tension value was 38 mN/m for Vmh2 and 35 mN/m for PAC3. However, the surface tension of a PAC3 solution at the same concentration as Vmh2 (0.14 mg/mL) was significantly higher (44 mN/m) than that of Vmh2; thus, Vmh2 can be considered a more efficient surface-active protein than PAC3.

### 2.5. Surface Adhesion and Wettability Change

**Figure 8:**
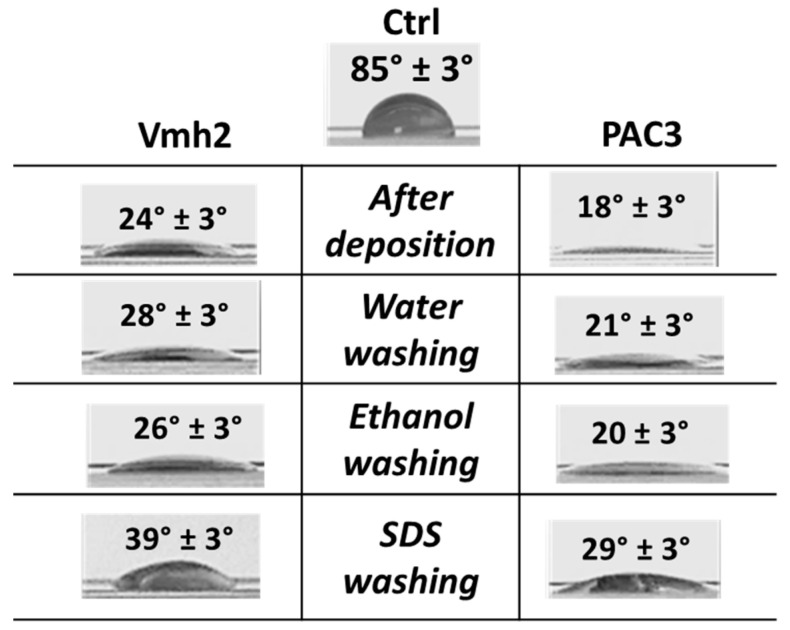
Water contact angle analysis. A total of 50 µL of protein solutions (0.1 mg/mL) was deposited on a polystyrene surface, dried out at RT, and differently washed as indicated. Then, a 10 µL water drop was deposited on the coated surface, and angles were estimated using Image J version: 1.8.0. Errors were calculated on the average of three deposited drops.

It is well known that HPBs can change the wettability of surfaces on which they are deposited [20]. In particular, the ability of PAC3 and Vmh2 to reverse the wettability of a hydrophobic surface, such as polystyrene, was tested. The proteins were deposited on the surface and air-dried; then, the surface was washed as reported in Figure 8, and the contact angle of a water drop was calculated. Both proteins formed stable layers, changing the wettability of the polystyrene surface and maintaining strong adhesion even after SDS washing.

## 3. Discussion

It is worth noting that PAC3 from *A. sclerotigenum* behaves very similarly to HPBs [15,16], even though it does not show the sequence features of that family. Indeed, mass spectrometry studies reported in this paper indicate aminoacidic sequence similarity with an unknown fungal protein. The sequence of Sap-Pc from *Penicillium chrysogenum*, a presumed HPB based on its functional properties, appears to be that of an unknown protein lacking the typical cysteine pattern of HPBs [21]. Both proteins, PAC3 and Sap-Pc, are surface-active and have good emulsification ability. They change their secondary structures, forming aggregate structures positive to the ThT tests, over time and depending on the protein concentration. Analysis by using AFM of PAC3 aggregates shows the rodlet structure, typical of class I HPB, while the morphology of Sap-Pc fibrils looks like a “pearl necklace”, originated by an array of interacting aggregates. All of these findings open up new perspectives to discover fungal surface-active proteins beyond the members of the HPB family. In this paper, the characteristics of PAC3 have been compared to those of a canonical HPB, Vmh2.

The first important aspect is the spontaneous formation of large amyloid fibers. To the best of our knowledge, this is the first evidence of giant fibers obtained using a whole protein without the need for harsh conditions or a long incubation time. Indeed, until now, giant fibers have been obtained only upon an engineered control of amyloid fibril assemblies, such as using opportunely mixed protein hydrolysates [22,23,24,25,26,27,28,29,30], or specific deposition procedures designed to apply external constraints to direct the assembly of protein nanofibrils, such as microfluidics [31,32] and electrospinning [33,34].

As regards the characterization of BE activities, our results indicated that both proteins are good BEs (E_24_: 70% Vmh2 and 60% PAC3 at 0.1 mg/mL), better or comparable to that of other fungal surfactant proteins. Indeed, the E_24_ value of SapPC at the same concentration was 70%, and the E_24_ of the ceratoplatanins *At*Cp and *Th*Cp was 83% and 70%, respectively. Moreover, the emulsification ability of HFBII, a class II HPB characterized by Blesic et al. [18] using the same protocol, showed an emulsion index of 74% at a concentration of 1 mg/mL, a ten times higher concentration. PAC3 remarkably stabilized emulsions even at lower concentrations (20–50 µg/mL). Regarding the effect of pH, we observed a stronger dependence on the pH of the emulsion activity of PAC3 as compared to Vmh2, whose E_24_ does not change in the tested pH range. On the other hand, they are both negatively affected by salt concentration. In addition, the results showed that incubation at high temperatures has also a negative effect, but emulsification ability can be restored, as well as their secondary structures, when protein solutions are slowly cooled down. 

Analysis of the reduction in the surface tension of these proteins is affected by protein solubility and by the aggregation phenomena occurring at relatively high protein concentrations. For these reasons, a constant surface tension value was not reached (Figure 7), noting that Vmh2 possesses a more significant surface activity than PAC3. Compared with synthetic surfactants, Vmh2 showed similar results to Triton X-100^®^, a non-ionic surfactant that reaches tension values of 31 mN/m at 2 × 10^−4^ M [35]. As another example, SDS achieves a surface tension of 35 mN/m [36] at 3 × 10^−3^ M, a significantly higher concentration than that used for the studied proteins. 

However, Vmh2 caused weaker surface tension reduction compared with other surfactant proteins, e.g., class I HPB SC3p and class II HPB CTFH1, and decreased surface tension at values of 32 and 33 mN/m for a 0.1 mg/mL solution (6.8 × 10^−6^ and 2.8 × 10^−6^ M, respectively), and *Th*CP decreased surface tension until 36 mN/m at 0.01 mg/mL (8 × 10^7^ M) [37]. According to commonly used grouping, these fungal proteins are more efficient BEs than BSs. 

## 4. Materials and Methods

### 4.1. Fungal Culture Conditions 

*P. ostreatus* and *A. sclerotigenum* (MUT 4872) were maintained at 4 °C through periodic transfer on potato dextrose agar plates in the presence of 0.5% yeast extract or on agar plates in the presence of XNST30 (malt extract 3 g/L, yeast extract 3 g/L, sodium chloride 30 g/L, 10 g/L glucose, and 5 g/L peptone), respectively. Mycelium disks (10 mm diameter) were taken from the margin of actively growing colonies, inoculated in 1 L flasks containing 500 mL of potato dextrose broth (24 g/L) supplemented with 0.5% yeast extract (*P. ostreatus*) or XNST30 (*A. sclerotigenum*), grown in duplicate at 28 °C in shaken mode (180 rpm). 

### 4.2. Protein Purification

#### 4.2.1. Vmh2 

The protein was extracted and purified following the protocol of Gravagnuolo et al. [12]. Briefly, the lyophilized mycelia were washed with 2% SDS and then freeze-dried; upon lyophilization, it was treated with pure TFA, obtaining the raw extract. Then, the extract was solubilized in 60% ethanol and further purified through methanol-chloroform extraction to remove lipids. The protein was finally resuspended in a 60% ethanol solution. This purification protocol yielded 24 mg of protein per gram of mycelium. 

#### 4.2.2. PAC3

Purification was performed from the culture broth according to the protocol of Cicatiello et al. [15]. Briefly, the culture broth was stirred using a blender to produce foam. The recovered foam was incubated o.n. with 20% trichloroacetic acid and centrifuged (3000 rcf 60 min). Upon acid precipitation, the pellet was treated with pure TFA and suspended in 60% ethanol to obtain the raw extract. It was extracted in a mixture of water–methanol–chloroform. After centrifugation, the protein was recovered by removal of the liquid phases, treated with TFA for 20 min in a bath sonicator, dried again, and dissolved in 60% ethanol to obtain 20 mg of protein per liter of culture. 

Before any experiment, both proteins were dried out, treated with TFA, and solubilized in aqueous buffer (50 mM Tris HCl pH 8 unless otherwise stated).

### 4.3. MALDI-TOF Analysis of PAC3

A total of 100 µg of extracted PAC3 protein was hydrolyzed with trypsin by using the following procedure. The reduction reaction was performed by adding 20 µL of 100 mM dithiothreitol and 6M urea at 60 °C for 1 h. A total of 20 µL of 120 mM iodoacetamide was added, and the reaction was conducted for 45 min at room temperature in the dark. A total of 500 µL of methanol was used to perform protein precipitation. The solution was centrifuged for 15 min at 13,680 rcf, and the pellet was dried under vacuum and then suspended in 50 µL of 10 mM ammonium bicarbonate (AMBIC) with 2 µL of trypsin (1 µg/µL in 10 mM AMBIC) at 37 °C for 15 h. A total of 0.5 µL of the peptide mixture was deposited on a MALDI plate with an equal volume (1/1, vol/vol) of a solution of α-cyano-4-hydroxycinnamic acid (20 mg/mL) dissolved in 70:30 acetonitrile:55 mM citric acid in water. MALDI-MS experiments were performed on a 4800 MALDI-TOF ABSciex equipped with a nitrogen laser (337 nm). Spectra were acquired in the reflector-positive mode by using a 400–4000 (*m*/*z*) mass range. Laser power was set to 4200 V for MS spectra acquisition. Each spectrum represents the sum of 3000 laser pulses from randomly chosen spots per sample position by covering the entire spot. Calibration was performed by using an AB SCIEX calibration mixture (Monoisotopic (M + nH) n+:904.46 Da des-Arg-Bradykinin, 1296.68 Da Angiotensin I, 1570.67 Da Glu-Fibrinopeptide B, 2093.08 Da ACTH (clip: 1–17), 2465.19 Da ACTH (clip: 18–39), 3657.92 Da ACTH (clip: 7–38)). The data were elaborated using the Data Explorer 5.1 software (Applied Biosystems, Foster City, CA, USA), and the *m*/*z* values were reported as monoisotopic masses. The MS/MS spectra were also recorded in positive mode, and air was used as collision gas with a medium pressure of 10–6 Torr for CID experiments. The Glu-Fibrinopeptide peptide (1570.67 Da) was used to calibrate MS/MS acquisition [38,39]. 

### 4.4. Confocal Laser Microscopy 

Image acquisitions were performed with a confocal laser scanning microscope (CLSM) (LSM700-Zeiss, Oberkochen, Germany) equipped with an Ar laser (488 nm) and a He–Ne laser (555 nm). Images were obtained using a 20×/0.8 objective. The excitation/emission maximum for the ThT dye is 405 nm. Emulsions were visualized by placing samples on a glass slide and letting them dry at room temperature.

### 4.5. Scanning Electron Microscopy

Scanning electron microscopy (SEM) images were obtained by using a FEI Nova NanoSEM 450 at an accelerating voltage of 1–5 kV with Everhart–Thornley detector (ETD) and through lens detector (TLD) at high magnification (FEI, Austin, TX, USA). Samples were prepared for SEM analyses by depositing proteins onto Al or polystyrene substrates and sputter coating them with a thin layer of Au-Pd (Sputter Coater Denton Vacuum Desk V).

### 4.6. Emulsification Index Evaluation

Proteins were dissolved in different aqueous buffers and placed in a 5 mL glass vial, and 2 mL of Dectol (Decane:Toluene 65:35) as an emulsifying agent was added [18]. This mixture was homogenized using an IKA T-10 Basic Ultra Turrax Homogenizer for 3 min in a 5 mL glass vial; then, the emulsion ability was reported in terms of emulsification index E_24_, calculated after 24 h by dividing the final height of the emulsion layer by the total height and multiplying by 100. The emulsification activity was investigated at different protein concentrations; additionally, it was tested by dissolving the proteins at different pH using different solution buffers (50 mM sodium phosphate at pH 6 and pH 7 and 50 mM Tris-HCl from pH 8 to 10). The effect of two different sodium chloride concentrations was tested when the protein was suspended in 50 mM Tris-HCl pH 8. Errors on all of the E_24_ evaluated were calculated as the deviation standard for at least two biological replicates and three technical replicates.

### 4.7. Effect of Temperature

The effect of temperature was investigated by incubating protein solutions (0.05 mg/mL in 50 mM sodium phosphate pH 7.5) at 95° C for 20 min. Emulsions with Dectol were performed right after heating and after cooling down proteins at room temperature for 60 min.

### 4.8. Protein Hydrolysis

Hydrolysis with proteinase K was performed after a pretreatment of the sample. A solution of 0.05 mg/mL of the sample was first incubated with 5 mM of dithiothreitol (DTT) for 10 min at 60 °C. Then, after the solution had cooled, protease K and CaCl2 were added to a final concentration of 1 mg/mL and 0.5 mM, respectively, and the solution was incubated at 40 °C for 1 h. 

### 4.9. Circular Dichroism

CD spectra were recorded on a Jasco J1500 spectropolarimeter (Jasco Corporation), (Jasco Corporation, Cremella (LC), Italy) equipped with a Peltier thermostatic cell holder in a quartz cell (0.1 cm light path) from 190 to 250 nm. Spectra were acquired before and right after the thermal shock at 95 °C for 20′, according to the emulsification tests, under continuous nitrogen flush.

Final spectra (60′ at room temperature) were obtained by averaging six scans using a bandwidth of 1 nm, a step width of 0.5 nm, and a velocity scan of 100 nm/min.

### 4.10. Surface Tension Measurement 

Drop collapse consists of depositing a sample drop onto a hydrophobic surface (polystyrene) and then evaluating the collapsing area. The BS solution should spread on the surface more extensively than water in image analysis on the photos using Image J version: 1.8.0 (Rasband, W.S., ImageJ, U.S. National Institutes of Health, Bethesda, Maryland, USA) [40].

The surface tension γ of the extracted protein solutions was measured with a Sigma 70 tensiometer (KSV, Stockholm, Sweden) using the Du Noüy ring method as described elsewhere (Huh et al., 1975). γ was correlated with the force required to raise the ring from the surface of the air/liquid interface. Aliquots of the proteins suspended in 50 mM pH 8 Tris-HCl buffer were prepared at different concentrations; each sample was filtered with a 0.22 μm filter before testing. A total of 7 mL of the sample was added to the vessel and allowed to equilibrate at least 5 min prior to measuring the surface tension. A 50 mM Tris-HCl buffer with a surface tension of 73 mN/m was used as a control. The results were the average of at least three different measurements. 

### 4.11. Surface Adhesion and Wettability Change 

To test the ability of the proteins to adhere to and change the wettability of surfaces, they were deposited on a polystyrene surface, which is highly hydrophobic. A total of 50 µL of proteins was deposited and air-dried, and then the surface was washed with water, 60% ethanol, or 2% sodium dodecyl sulfate (SDS). The contact angle of a drop of water on the surface was calculated after every washing step. The wettability change was evaluated by calculating the contact angle through image analysis on the photos using Image J version: 1.8.0 [40].

## 5. Conclusions

Besides highlighting the efficiency of Vmh2 and PAC3 as BEs, this paper poses questions about the peculiarity of the HPBs protein family, since other fungal proteins share functional characteristics with this protein family even though they do not share the same sequence features (i.e., the conserved eight cysteines pattern). Among the typical characteristics of the herein-studied proteins, the most remarkable is the spontaneous and easy formation of giant amyloid fibers. This ability could be exploited in the controlled formation of micro- and macro-fibrils through a microfluidic spinning approach or core–shell electrospinning methods [31,41]. The final aim would be the production of functional fibers by using both native and recombinant proteins, also fused with other peptides/proteins of interest. Moreover, the surface-active characteristics shown by the analyzed proteins widen their potential applications. In this context, the use of proteinaceous surfactants could tackle the challenge of large-scale production of BSs and BEs. Indeed, proteinaceous surfactants can be recombinantly produced on low-cost raw materials/wastes as growth substrates and purified using easy processes, leading to cost-effective processes and high production yields [2].

## Supplementary Materials

The following supporting information can be downloaded at: https://www.mdpi.com/article/10.3390/ijms241813843/s1.
