# Supplementary material for: Evidence of Small Fungal Cysteine-Rich Proteins Acting as Biosurfactants and Self-Assembling into Large Fibers
Source: Int J Mol Sci. 2023 Sep 8;24(18):13843. doi: 10.3390/ijms241813843 (PMC10531366; doi:10.3390/ijms241813843)
Supplement: Supplementary file 1 [file ijms-24-13843-s001.zip › ijms-2550806-SI.pdf]

Evidence of small fungal cysteine-rich proteins acting as biosurfactants and self-assembling into large fibers.

Rossana Pitocchi[1], Ilaria Stanzione[1], Anna Illiano[1], Angela Amoresano[1], Oreste Tarallo[1], Paola Cicatiello[1]*, Alessandra Piscitelli[1], Paola Giardina[1]

[1] Department of Chemical Sciences, University of Naples Federico II, Via Cintia, 80126 Naples, Italy.

* Correspondence: paola.cicatiello@unina.it; Tel. +3908174327

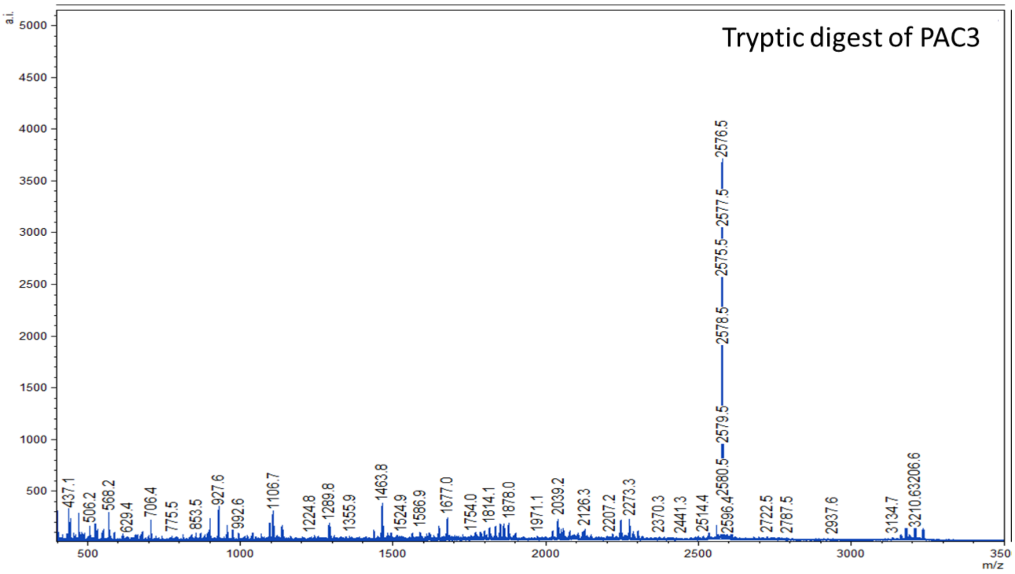

Figure S1. MALDI-MS spectrum of tryptic peptides of PAC3 acquired in the range 400 - 3500 m/z.

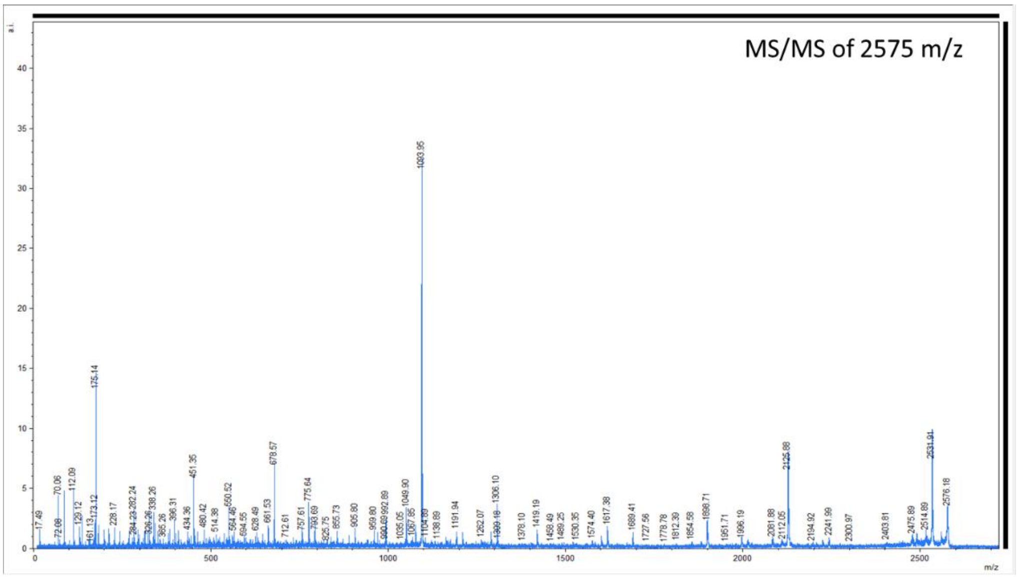

Figure S2. MALDI MS-MS fragmentation spectrum of the 2575 m/z PAC3 tryptic peptide (named peptide 1).

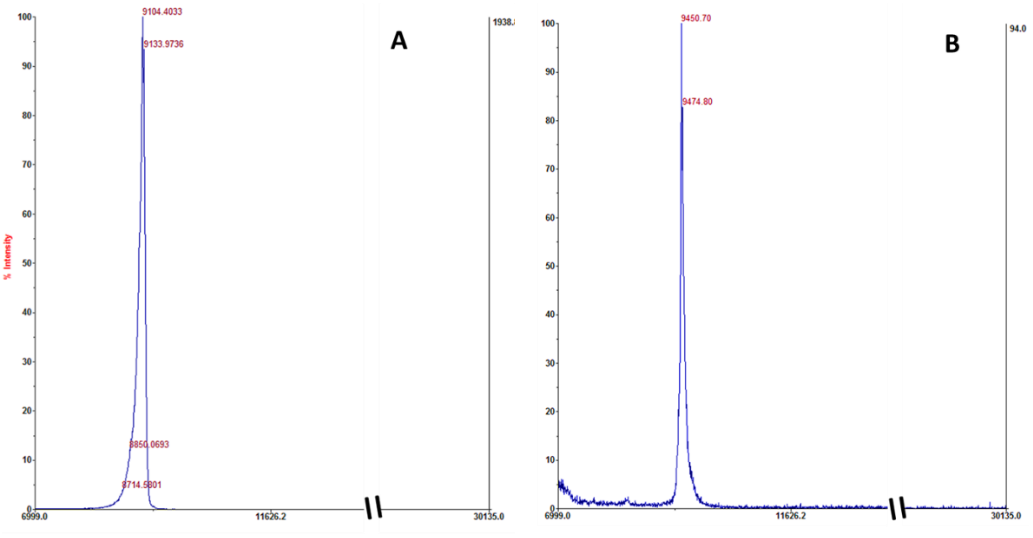

Figure S3. MALDI-TOF spectrum of PAC3 protein. The spectrum A is related to the untreated PAC3, while the spectrum B is related to the reduced and carbamidomethylated PAC3.

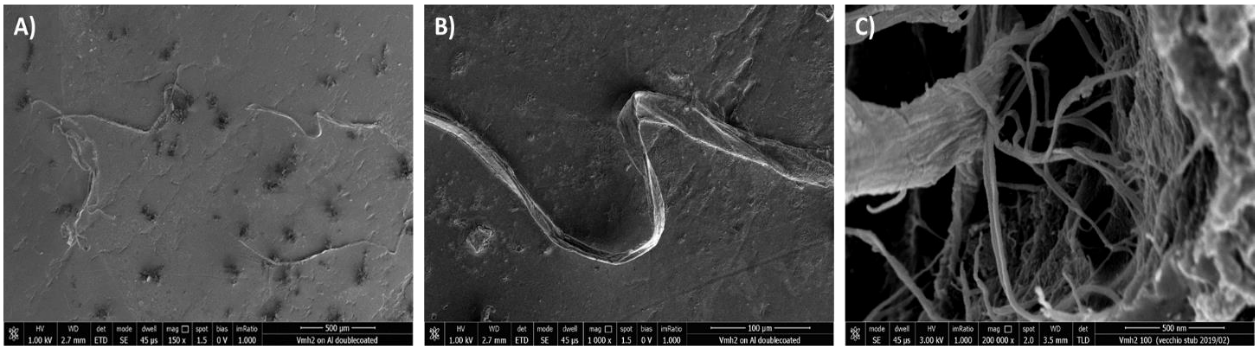

Figure S4. SEM images of Vmh2 fibers at different magnification (A, B, C, at 150X, 1000X, 200000X respectively). 20 µl of protein solutions were deposited on the surfaces, air dried and Au-Pd coated prior visualization.

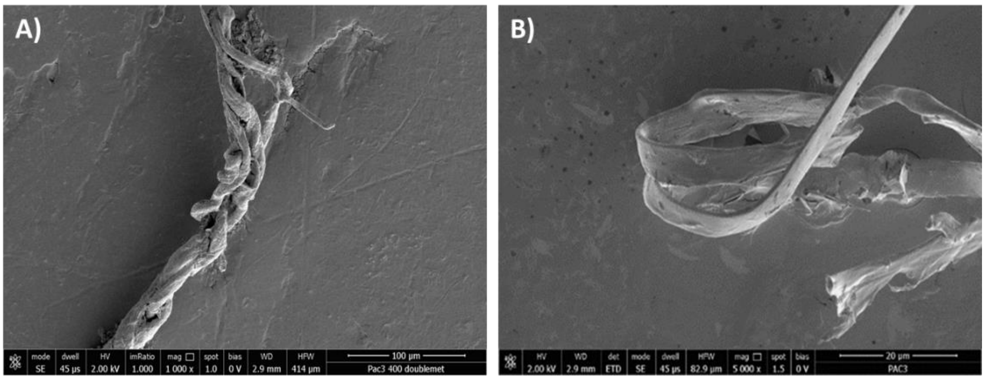

Figure S5. SEM images of PAC3 fibers at different magnification (A, B, at 1000X, 5000X respectively). 20 µl of protein solutions were deposited on the surfaces, air dried and Au-Pd coated prior visualization.

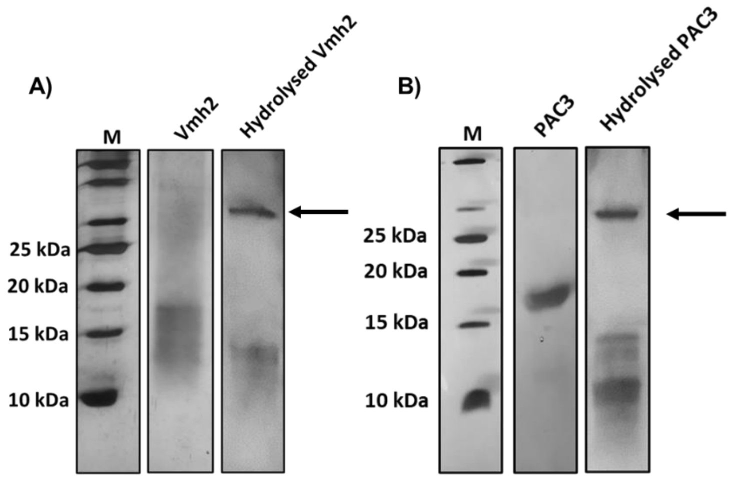

Figure S6. SDS-PAGE before (first lane) and after hydrolysis (second lane) of Vmh2 (A) and Pac3 (B) with Proteinase K, indicated in the figure by the arrows.

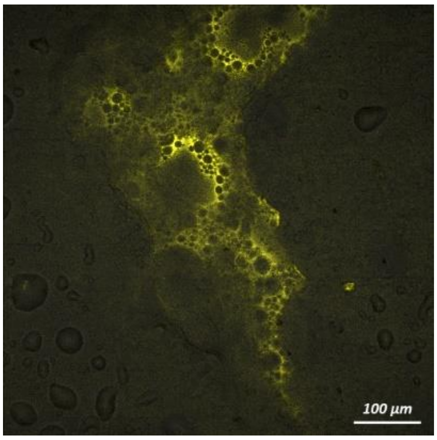

Figure S7. Emulsions of PAC3 solution (0.05 mg/ml) containing 3µM ThT mixed with Dectol (2:1 v/v), analysed by confocal laser microscopy. Image was acquired depositing the foam on a glass slide and drying at RT.
